# Dynamic Changes in Ion Channels during Myocardial Infarction and Therapeutic Challenges

**DOI:** 10.3390/ijms25126467

**Published:** 2024-06-12

**Authors:** Tongtong Song, Wenting Hui, Min Huang, Yan Guo, Meiyi Yu, Xiaoyu Yang, Yanqing Liu, Xia Chen

**Affiliations:** 1 Department of Pharmacology, College of Basic Medical Sciences, Jilin University, Changchun 130012, China; songtongtong@jlu.edu.cn (T.S.); 17808065058@163.com (W.H.); huangmin22@mails.jlu.edu.cn (M.H.); yany.guo@outlook.com (Y.G.); 17860554155@163.com (M.Y.); yangxiaoyu199202@163.com (X.Y.); l1025273765@163.com (Y.L.); 2 Department of Anatomy, College of Basic Medical Sciences, Jilin University, Changchun 130012, China

**Keywords:** ion channels, myocardial infarction, potassium channels, calcium channels, sodium channels

## Abstract

In different areas of the heart, action potential waveforms differ due to differences in the expressions of sodium, calcium, and potassium channels. One of the characteristics of myocardial infarction (MI) is an imbalance in oxygen supply and demand, leading to ion imbalance. After MI, the regulation and expression levels of K^+^, Ca^2+^, and Na^+^ ion channels in cardiomyocytes are altered, which affects the regularity of cardiac rhythm and leads to myocardial injury. Myocardial fibroblasts are the main effector cells in the process of MI repair. The ion channels of myocardial fibroblasts play an important role in the process of MI. At the same time, a large number of ion channels are expressed in immune cells, which play an important role by regulating the in- and outflow of ions to complete intracellular signal transduction. Ion channels are widely distributed in a variety of cells and are attractive targets for drug development. This article reviews the changes in different ion channels after MI and the therapeutic drugs for these channels. We analyze the complex molecular mechanisms behind myocardial ion channel regulation and the challenges in ion channel drug therapy.

## 1. Introduction

The normal function of the heart relies on the generation and spread of action potentials [1,2,3]. Action potentials reflect the sequential activation and inactivation of in- and outward current-carrying ion channels [1]. In different regions of the heart, the waveforms of action potentials vary due to differences in Na^+^, Ca^2+^, and K^+^ channel expression, and these differences contribute to the generation of normal rhythm [1,4]. Genetic or acquired diseases lead to the abnormal expression or function of the ion channels in cell membranes, which can cause life-threatening arrhythmias [1,5].

When MI occurs, it causes changes in the function of ion channels in the myocardium [6,7]. The surviving myocardial cells in the infarct scar and the marginal zone have poor electrical coupling, a slow conduction of electrical signals, a unidirectional block, and re-entrant conduction, which induces the occurrence of sustained ventricular tachycardia [8]. The remodeling of other important components within and outside the infarct further adds to the complexity of the post-MI matrix, including cellular ionic currents, gap junctions, and the remodeling of intramyocardial nerve fibers [8,9].

The development of high-throughput patch-clamp [10] and Flexstation [11] technology has promoted the detection of ion channels and the development of targeted drugs [12]. The ion channel is a type of transmembrane protein in biological cells. It is generally a water-filled pore enclosed by several protein subunits. Different ion channels can be opened under different stimuli to allow specific ions to pass through the pore [13]. Ion channels play a central role in many physiological processes, and they represent a target class with great potential for intervention in various disease states [14,15]. For example, ion channels affect the intracellular Ca^2+^ concentration either directly, by allowing Ca^2+^ flux, or indirectly, by modulating membrane potential. This, in turn, regulates multiple functions, including muscle contraction, hormone secretion, and gene transcription [16,17].

This article provides a comprehensive review of the alterations in ion channels and their treatments following MI.

## 2. Ion Channel Changes after MI

### 2.1. Ion Current Remodeling after MI

Changes in the pathological environment during MI can lead to the remodeling of myocardial ion currents [18]; the changes that occur in these currents in different regions of myocardial tissue are heterogeneous. The remodeling of surviving epicardial and boundary muscle cells after MI leads to electrical conduction abnormalities, and surviving endocardial Purkinje fibers may be an important trigger [19]. In addition, days to weeks after MI, non-infarcted myocardial, including the peri-infarct boundary area, also undergo significant ion current remodeling [20].

During the acute phase of myocardial ischemia (a few minutes to 2–4 h after reduced coronary blood flow), the resting membrane potential of ischemic cardiomyocytes is significantly depolarized, in part due to K^+^ loss and intracellular acidosis, resulting in extracellular K^+^ accumulation in the ischemic area. The depolarization of the resting membrane potential inhibits the upstroke and amplitude of the action potential by reducing the fast Na^+^ current (INa). This leads to a prolonged refractory period due to a slow recovery of the inactivated Na^+^-gated channel. Additionally, the activation of ATP-dependent K^+^ channels (IK (ATP)) was found to shorten the action potential duration (APD) [21]. Elevated Ca^2+^ and H^+^ levels, an accumulation of amphiphilic lipid metabolites, and a dephosphorylation of the gap junction protein Connexin43 (Cx43) over 4–15 min of ischemia reduce intercellular electrical coupling [22], and these changes lead to impaired impulse conduction during acute ischemia.

During the subacute and chronic stages of MI, surviving Purkinje fibers demonstrate a depolarizing resting potential. This has been associated with a significant decrease in the density of inward-rectified K^+^ currents (IK1), T-type and L-type Ca^2+^ currents (ICaT and ICaL), and volt-dependent transient outward currents (Ito1) [23,24,25]. The surviving epicardial cells in the infarct boundary area have shown decreased excitability and resting potential, and a decreased upstroke velocity and action potential amplitude [26]. The pathological redistribution of CX43 protein and decreased gap junction conductivity were observed in boundary-zone surviving cardiomyocytes [27]. These changes result in pulse conduction becoming abnormal, slow, and change in direction [28]. With infarct healing, the resting potential, action potential amplitude, and upsurge velocity of surviving epicardial myocytes were found to return to normal within 2 weeks, but the action potential duration decreased further. By 2 months, the action potential had also returned to normal [29]. The peak and maximum rate of action potential depolarization in ventricular myocytes in non-infarcted areas after MI were not significantly different from those in normal control cells, but APD was significantly increased during repolarization. One mechanism by which APD increases after MI is a decrease in the voltage-gated K^+^ channel (Kv) currents responsible for ventricular cell repolarization, including Ito, IKslow1, and IKslow2. This effect was associated with the decreased expressions of the Kv1.5, Kv2.1, Kv4.2, and Kv4.3 proteins [30,31]. At the same time, the increase in APD promotes Ca^2+^ inflow [31].

### 2.2. Ca^2+^ Channels after MI

**Figure 1:**
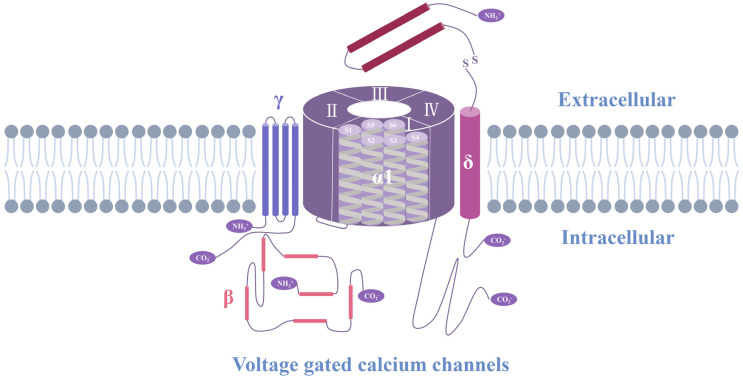
Voltage-gated Ca^2+^ channel structure. The subunit consists of four homologous domains (I–IV) [35,36,37,38,39]. The five-subunit complex that forms high-voltage-activated Ca^2+^ channels is illustrated with a central pore-forming α1 subunit, a disulfide-linked glycoprotein dimer of α2 and δ subunits, an intracellular β subunit, and a transmembrane glycoprotein γ subunit [40]. Each domain contains six transmembrane helices (S1–S6) [41].

Ca^2+^ channels can be gated through voltage or ligand binding. Voltage-gated Ca^2+^ channels (VGCCs) are the main source of cardiac Ca^2+^ influx and affect excitation–contraction coupling in myocardial tissue [32]. VGCCs are composed of α1, α2, δ, β, and γ subunits (Figure 1) [33]. The α subunit is the largest and ranges in size from 190 to 250 kDa. It contains a conduction pore, voltage sensor, and gate control mechanism, as well as most of the known sites for regulation through secondary messengers, drugs, and toxins. The α1 VGCC subunit is organized into four homologous domains (I–IV) with six transmembrane segments in each domain. The S4 segment serves as the voltage sensor. The ion conductance and selectivity are determined by the pore ring between transmembrane segments S5 and S6 in each domain. The cellular β and α2δ subunits, which are cross-linked by a transmembrane disulfide bond, are components of most types of Ca^2+^ channels [34]. Ca^2+^ channels can be divided into five types according to their activation characteristics: L-, P/Q-, N-, R-, and T-type. Among them, the L- and T-type Ca^2+^ channels are the main ones that affect the pathophysiological process of the heart.

L-type Ca^2+^ channels (LTCCs) are activated by depolarization at each action potential [42]. After MI in rats, a decrease in LTCC density was detected using a whole-cell patch clamp [43]. Impaired LTCC function further leads to a reduced sensitivity of cardiomyocytes to β-adrenergic stimulation [44]. Using super-resolution scanning patch-clamp techniques, Jose L. Sanchez Alonso et al. detected that functional LTCCs were dislocated to the sarcolemmal surface in failing cardiomyocytes and that these repositioned LTCCs exhibited a higher open probability (Po) and phosphorylated state. This phenomenon may be associated with Ca^2+^/calmodulin-dependent protein kinase II (CaMKII) activity [45]. Janet R. and others revealed that guanosine triphosphatase (GTPase) Rad protein expression in the heart of family members has a significant effect on the damaged heart’s LTCC current [46]. They also observed that inhibiting Rad protein can improve myocardial systolic function after MI [46]. Previous studies have shown that the Cav1.3 channel is expressed in atria and pacemaking cells in the heart [47].

T-type Ca^2+^ channels (TTCCs) are low-voltage-gated channels with fast kinetics; their activation does not require strong depolarization, and they have a relatively transient activation state [48]. Cardiac T-type channels are composed of two major types, Cav3.1 (α1G) and Cav3.2 (α1H), both of which can be induced in diseased and injured myocardium. Le Quang et al. reported that the knockout of the cardiac T-type Ca^2+^ channel subunit Cav3.1 adversely affected remodeling after MI, which mainly manifested as an exacerbation of arrhythmias and a decrease in myocardial contractility [49]. TTCCs have been proven to play a vital role in the process of pathological cardiac hypertrophy by increasing the Ca^2+^ flow induced through Cav3.2 calcineurin-related hypertrophy signaling [50].

The transient receptor potential cation subfamily V member 1 (TRPV1) and member kcnk2 (TRPV2) channels are also reported to allow the passage of Ca^2+^ current. After myocardial ischemia/reperfusion injury (I/R), a study found that cardiomyocyte viability decreased, cell apoptosis increased, and intracellular Ca^2+^ concentration rose. The upregulation of TRPV1 decreases cardiomyocyte viability and increases apoptosis and intracellular Ca^2+^ concentration, while the downregulation of TRPV1 further prevents I/R damage [51]. Other studies have shown that downregulated TRPV2 can significantly inhibit ROS production and Ca^2+^ concentration in cardiomyocytes. The inhibition of TRPV2 can decrease the apoptosis rate of cardiomyocytes treated with I/R and reduce heart damage [52].

### 2.3. K^+^ Channels after MI

K^+^ currents determine the resting membrane potential and control the repolarization of cardiomyocytes. K^+^ channels have transmembrane helices (TMs) that span the lipid bilayer. The K^+^ channel pore is composed of four, usually identical, subunits that surround the central ion conduction pathway in a tetrameric symmetrical arrangement. Each subunit contains two fully transmembrane alpha helices, called the inner and outer helices (closest to the ion pathway and the membrane, respectively), as well as a slanted pore helix that penetrates halfway through the membrane and directs its C-terminal negatively charged end toward the ion pathway [53]. Based on structure and function, the channels are classified into three major categories: voltage-gated (Kv) (six TMs), inward-rectifying (Kir) (two TMs), and series pore-domain (K2P) (four TMs) [54]. In addition, ligand-gated (K ligand) have either two or six TMs and are stimulated by various signaling molecules [55]. K^+^ channel dysfunction is associated with intracellular signaling, metabolism, remodeling, and arrhythmogenesis in many cardiovascular diseases and is essential for cardiac electrophysiology [56].

The electrical activity of cardiomyocytes is regulated by ATP-dependent K^+^ channels (KATP), which are important metabolic sensors with the ability to significantly shorten action potentials. It has been found that unimpaired K^+^ channel components are essential for ischemic preconditioning (IPC). However, in one study, the action potential of myocardial cells in the infarct border zone was prolonged compared with the sham operation group [57]. The Twik-associated K^+^ channel (TREK-1) is a two-pore-domain K^+^ channel belonging to the K2P channel family. Early studies have shown that TREK-1 protects against ischemia-induced neuronal damage. In recent years, it has also been found to play a key role in the remodeling process of MI. Electrocardiogram (ECG) analyses have revealed a prolonged QT interval in TREK-1 knockout mice, and TREK knockout cardiomyocytes exhibited a prolonged Ca^2+^ transient duration associated with a prolonged action potential duration (APD) [58]. Xuwen Zhai et al. [59] showed that Kir channel expression was significantly downregulated after MI, which in turn induced ventricular arrhythmias via CaMKII signaling.

It is worth mentioning that the downregulation of K^+^ channels in cardiomyocytes after MI is closely related to the influx of Ca^2+^. The decrease in K^+^ channel expression after MI prolongs the action potential duration and leads to a large influx of Ca^2+^ [60]. Recently, it has been found that andrographolide protects against isoproterenol-induced MI in rats through the inhibition of L-type Ca^2+^ and an increase in cardiac transient outward K currents^+^ [61]. K_ir2.1_ is encoded by the *KCNJ2* gene, which plays an important role in maintaining cell resting membrane potential (RMP), regulating cell excitability, and participating in various physiological processes. Dysfunctional K_ir2.1_ channels can disrupt the normal electrical activity of the heart, leading to potentially life-threatening arrhythmia. K_ir2.1_ channels interact with a variety of regulatory proteins, including protein kinase A (PKA), protein kinase C (PKC), and phosphatidylinositol-4,5-diphosphate (PIP2). For example, the opening probability of recombinant K_ir2.1_ and K_ir2.3_ is inhibited by PKA- and PKC-mediated phosphorylation. Additionally, PIP2 is a key lipid component of the plasma membrane that acts as a positive regulator of the K_ir2.1_ channel by binding to specific sites within the channel structure, thereby modulating the channel and enhancing its probability of opening [62].

### 2.4. Na^+^ Channels after MI

Cardiac Na^+^ channels are transmembrane proteins distributed in atrial and ventricular myocytes and Purkinje fibers. There are nine subtypes of Na^+^ channels: Nav1.1 to Nav1.9. Nav1.1, Nav1.2, Nav1.3, and Nav1.6 primarily function in the central nervous system. Nav1.4 and Nav1.5 act in the skeletal muscle and heart, respectively. Nav1.7, Nav1.8, and Nav1.9 are mainly found in the peripheral nervous system [63]. The Nav channels are made up of a pore-forming α-subunit and one or more auxiliary β-subunits. These β-subunits regulate voltage dependence, gating kinetics, and channel density. They fold as an extracellular IgG domain with a single transmembrane domain. The α-subunits are pseudo tetramers that are roughly 2000 amino acids in length and contain four transmembrane domains (DI–DIV), each of which contains six membrane-spanning α-helices (S1–S6). Together, the four domains form the pore of the channel, which opens to allow an inward Na^+^ current [64]. The action potentials and subsequent excitation–contraction coupling in cardiomyocytes are triggered by a significant and rapid influx of Na^+^ through these channels [65]. The rate and uniformity of action potential rise and spread in cardiac tissues are controlled by the rapid activation of voltage-gated Na^+^ channels [66]. In addition, the interactions of cardiac Na^+^ channels with other proteins may promote channel activity and membrane expression [67].

The expression of Na^+^ channels is involved in the regulation of biological electrical conduction velocity after MI. Ruben Coronel et al. found that Nav1.4 expression leads to an increase in longitudinal but not transverse conduction velocity [68] in the surviving epicardial layer of 1-week-old canine MI. The Nav1.5 current plays a crucial role in cardiac electrical conduction and arrhythmia risk. Upregulating Nav1.5 reduces the risk of arrhythmia after MI [69]. Studies have shown that Nav1.5 is regulated by the Src family tyrosine kinase Fyn through the phosphorylation of tyrosine residues. The regulation of the inactivation properties of the Na^+^ channels is due to the direct phosphorylation of tyrosine residues on the channel. The tyrosine phosphorylation of these Na^+^ channels is associated with a hyperpolarized shift in the steady-state inactivation, which leads to a reduction in the number of available channels for generating action potentials. Tyrosine kinases may also be activated through pathological states associated with cardiac ischemia and reperfusion injury and infarction-induced left ventricular remodeling [70].

Cardiac Na^+^ channel function is affected by a variety of proteins [71]. Na^+^ channel protein beta 1–4 subunits (encoded by genes *SCN1B*–*SCN4B*) and their respective alpha subunit (encoded by gene *SCN5A*) interactions affect the density of the Na^+^ channels and dynamics [72,73,74]. Ankyrins, fibroblast growth factor homologous factor 1B, calmodulin, caveolin-3, Nedd4-like ubiquitin-protein ligases, dystrophin, syntrophin, glycerol 3-phosphate dehydrogenase 1-like protein (GPD1L), and RAN guanine nucleotide release factor (MOG1) can directly bind to Nav1.5 and regulate Na^+^ channel transport, expression, and gating [75,76,77]. In addition, the density and kinetics of Na^+^ channels are regulated by phosphorylation and glycosylation as well as temperature [78].

## 3. Ion Channels in Cells of Myocardium

### 3.1. Cardiomyocytes

The rhythm of the heart originates from the electrical impulses generated by the regular opening and closing of ion channels in individual cardiomyocytes. These impulses spread throughout the myocardium, creating electrical waves that encompass the entire heart. The regularity of these waves is crucial, as these waves transmit the signal for myocardial contraction and drive the heart to pump blood to the brain and other vital organs [79].

After the occurrence of MI, myocardial cells in the areas of K^+^, Ca^2+^, and Na^+^ ion channels have varying degrees of damage [18]. In the early stage of myocardial ischemia, the cytosolic Na^+^ concentration is increased; in the late stage, the Na^+^ influx caused by Na^+^–hydrogen exchange (NHE) due to acidification further increases the intracellular Na^+^ content. With the increase in cytosolic Na^+^ content, Na^+^ efflux and Ca^2+^ influx are increased through a Na^+^/Ca^2+^ exchanger (NCX). The influx of Ca^2+^ ions through LTCCs results in intracellular Ca^2+^ overload, one of the adverse consequences of myocardial electrical activity [80].

Under normal conditions, LTCCs regulate Ca^2+^ release and maintain the electrical function of cardiomyocytes. When MI occurs, the myocardial membrane is depolarized, and LTCCs are activated to cause a small amount of Ca^2+^ ion to flow into the cytoplasm. The Ca^2+^ ion entering the cytoplasm triggers the opening of Ca^2+^ release channels in the sarcoplasmic and sarcoplasmic reticula and leads to intracellular Ca^2+^ overload [81]. Kai Huang et al. examined the mRNA expression levels of Ca^2+^ channel subunits after MI and found that the expression of the α1c and β2c subunits of the rat ventricular Ca^2+^ channel was significantly reduced, while the expressions of the α2/δ subunits were unchanged; this resulted in a reduced Ca^2+^-induced Ca^2+^ release (CICR) in the cells and myocardial systolic dysfunction [82].

The density of Ito K^+^ channels in epicardial cardiomyocytes is much higher than that in the endocardium, which leads to significant differences in the rate of the phase I repolarization of the action potential in cardiomyocytes between different myocardial layers. This is the main mechanism of the transventricular dispersion of repolarization (TDR). Existing research shows that the TDR expansion induced by ischemia increases the incidence of arrhythmia [83]. To a certain extent, the inhibition of epicardial Ito K^+^ channels can synchronize the repolarization rate of endocardial and epicardial cardiomyocytes and reduce the risk of arrhythmia [84]. Cardiomyocytes also express a large-conductance Ca^2+^-activated K^+^ channel (BKCa) in the mitochondrial inner membrane, which plays a central role in protecting the heart from ischemic injury [85]. It was found that BKCa may increase the Ca^2+^ retention capacity by regulating the mitochondrial Ca^2+^ pump, thereby allowing greater Ca^2+^ uptake during ischemia. In addition, blocking BKCa channels can enhance ROS production, thereby aggravating MI injury; however, it is still unclear exactly how this regulates ROS production [86].

### 3.2. Cardiac Fibroblasts

Cardiac fibroblasts (CFs) are the main effector cells in the process of MI, and the differentiation of fibroblasts into myofibroblasts is essential for the initial healing response to MI injury [87]. Myofibroblasts produce large amounts of extracellular matrix proteins, such as periostin, collagen, and fibronectin, which help maintain the structural and functional integrity of the left ventricle. However, excessive myofibroblast activation can lead to pathological fibrosis [88]. Intracellular ion oscillations play an important role in fibroblast action and myofibroblast contraction and trigger oxidative stress or downstream ion-dependent pathways involved in pathological cardiac remodeling [89].

**Figure 2:**
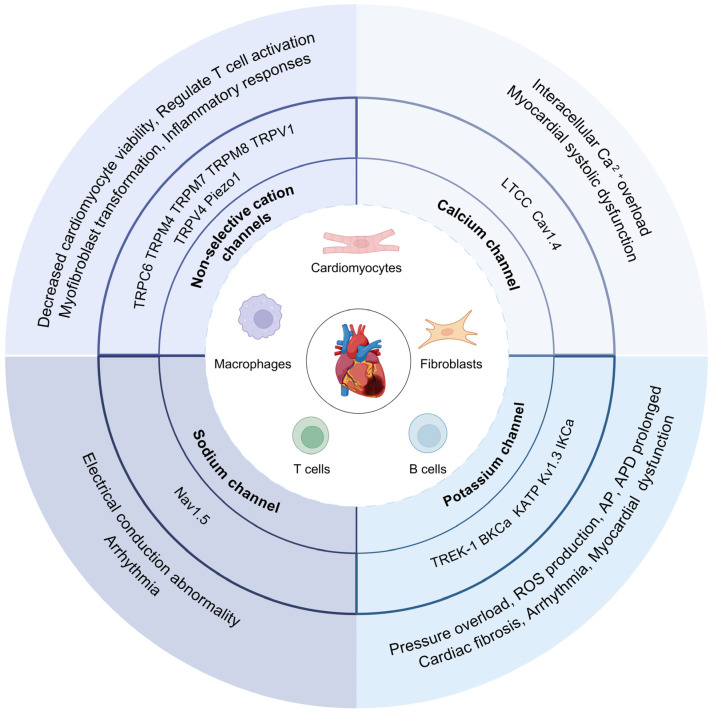
Ion channel subtypes involved in ion current remodeling after myocardial infarction. LTCC: L-type Ca^2+^ channel, Cav1.4: Ca^2+^ voltage-gated channel 1.4, TREK-1: TWIK-related K^+^ channel 1, BKCa: large-conductance Ca^2+^-activated K^+^ channel, KATP: ATP-sensitive K^+^ channel, Kv1.3: voltage-gated K^+^ channels 1.3, IKCa: intermedia-conductance Ca^2+^-activated K^+^ channel, AP: action potential, APD: action potential duration, Nav1.5: Na^+^ voltage-gated channel 1.5, TRPC6: transient receptor potential cation channel 6, TRPM4: transient receptor potential cation channel subfamily M member 4, TRPM7: transient receptor potential cation channel subfamily M member 7, TRPM8: transient receptor potential cation channel subfamily M member 8, TRPV1: transient receptor potential cation channel subfamily V member 1, TRPV4: transient receptor potential cation channel subfamily V member 4, PIEZO1: piezo-type mechanosensitive ion channel component 1.

With each contraction of the myocardium, the infarct scar is stretched, sometimes by 5–10% in the circumferential–longitudinal direction [90,91]. Accordingly, CFs in the infarcted region show increased mechanotransduction signaling activity. Currently, seven mechanosensitive ion channels are known to function in CFs: the K^+^ selective channels TREK-1 and KATP; the cation non-selective channels TRPC6, TRPM7, TRPV1, and TRPV4; and piezo-type mechanosensitive ion channel component 1 (PIEZO1) (Figure 2) [89,92]. These channels have been shown to have a direct role in the response of fibroblasts to mechanical stimuli. TREK-1 deletion in fibroblasts protects against pressure overload-induced cardiac function deterioration, which may be related to its regulatory effect on the JNK signaling pathway [93]. The activation of KATP channels can significantly attenuate the ischemia-induced differentiation of fibroblasts and inhibit the process of cardiac fibrosis [94]. TRPC6 was identified as a Ca^2+^ channel essential for myofibroblast transformation. The calcineurin–NFAT signaling axis mediates fibroblast differentiation. The overexpression of TRPC6 results in a 12-fold increase in NFAT activity in fibroblasts, while TGFβ induces only a 2-fold increase in NFAT activity [95]. TRPV1 and TRPV4 are also non-selective cation channels with high Ca^2+^ permeability and are involved in regulating the differentiation of cardiac fibroblasts into myofibroblasts [96,97]. In addition, it should be mentioned that intermediate-conductance KCa channels (IKCa) have also been identified to play important roles in regulating the biological functions of CFs. IKCa expression can promote fibroblast proliferation [98].

After MI, fibroblasts in scar tissue, especially myofibroblasts, can form electrical coupling with cardiomyocytes, and changes in the membrane potential of fibroblasts can cause muscle cell excitability and may lead to arrhythmia [8]. When the cardiomyocyte transmembrane potential (TMP) is negative for the fibroblast resting potential (e.g., at stage 4), the cardiomyocytes depolarize; in contrast, when it is positive (e.g., during AP peak and plateau), the cardiomyocytes repolarize [99].

In vitro studies have shown that gap junctions composed of Cx43 or Cx45 mediate direct electrical coupling between cardiac fibroblasts and co-cultured ventricular myocytes [100]. The coupled fibroblasts also exert a capacitive load on the cardiomyocytes in the boundary region by contributing additional membrane area to charge and discharge by the cardiomyocytes, effectively reducing the cardiomyocyte channel density. This, in turn, may significantly alter the cell membrane potentials of the heart muscle, such as the production and duration of action potentials. In addition, changes in the myocyte action potential waveform induced by this heterocellular coupling alter the transient properties of [Ca^2+^]i in myocytes, thereby affecting positive myocardial muscle strength by modulating the membrane potential-mediated Ca^2+^ transport pathway. Vice versa, Ca^2+^ flow from myocytes to myoblasts via gap junctions causes myoblasts to undergo repeated electrotonic depolarization in response to the myocytes’ action potential [101].

Wang et al. showed that fibroblast–myocyte coupling in scar tissue was robust enough to elicit cardiac excitation and arrhythmogenesis in vivo after fibroblast depolarization [8]. Dhanjal et al. used a porcine model of MI to investigate whether CFs play a role in slow conduction and arrhythmia inducibility 6 weeks post MI. Their data showed that CFs were primarily found in the isthmus of the re-entrant circuit. The high presence of CFs hinder conduction through the isthmus and increase the susceptibility to ventricular tachycardia (VT) [102].

### 3.3. Cardiac Immune Cells

Immune cells express various ion channels and transporters with an opening that allows the influx and efflux of ions across the plasma membrane (PM) or their release from intracellular organelles such as the endoplasmic reticulum (ER), mitochondria, or lysosomes [103]. Ion channels influence the development of infarction by affecting the function of immune cells. TRPM4 is a Ca^2+^ ion channel. Multi-omics analyses have revealed that TRPM4 deletion after MI in mice leads to enhanced pro-inflammatory effects, increases cardiac inflammatory responses in the first 24 h after MI, and induces earlier fibrosis at 72 h and chronic cardiac fibrosis and angiogenesis after 5 weeks [104]. TREM2 has anti-inflammatory effects during the inflammatory phase of MI. The administration of TREM2 to infarcted mice significantly improved myocardial function and the remodeling of the infarcted heart [105].

Macrophages are known to play critical roles in inflammation after MI [106]. The population of recruited macrophages quickly overwhelms the resident macrophages [107]. It has been demonstrated that resident macrophages can assist in electrical conduction in the atrioventricular node by forming gap junctions with cardiomyocytes. This may be related to ventricular arrhythmia induced by MI [108]. Yu-Dong Fei et al. found that macrophages accumulate and polarize into pro-inflammatory subtypes in MI border zones. They modulate the electrophysiological properties of cardiomyocytes via gap junctions and KCa3.1 activation, predisposing the heart to post-MI repolarization heterogeneity and arrhythmias. Macrophages and KCa3.1 ion currents are potential therapeutic targets against post-MI arrhythmias [109].

The Ca^2+^ channels Cav1.4, TRPM8, and PIEZO1 have been shown to regulate T cell activation and function [110,111,112,113]. The K^+^ channel Kv1.3 plays an important role in T cell activation, and Kv1.3 blockers have been found to inhibit T cell activation in a highly stimulus intensity-dependent manner [114]. Recent studies have found that the K^+^ channel K2P18.1 regulates the number of regulatory T cells (Tregs) [115]. In MI, T cells are activated and differentiate into different types of T cells with different roles [116]. CD8^+^ and CD4^+^ T cells are involved in the infarction process by initiating an adaptive immune response, whereas the prominent role of Tregs is to repair myocardial tissues by inhibiting the inflammatory process and activating fibrosis [117]. Existing studies have found that both Kv1.3 and KCa3.1 K^+^ channels may activate T lymphocytes and enhance cytokine secretion through the CaN/NFAT signaling pathway, which induces a microinflammatory response that triggers the onset and development of hypertension [118]. Furthermore, the effect of ion channel-induced T cell activation and differentiation on infarction needs to be further investigated.

Ion channels play a crucial role in regulating B cell activation and function. TRPV2 and TRPV5 are highly expressed in B cells and are important for B cell activation [119,120]. Additionally, Ca^2+^ channels are essential for the B cell immune response, as antigen binding to the BCR releases Ca^2+^ ions stored in the endoplasmic reticulum into the cytoplasm via ion channels. When the concentration of Ca^2+^ ions in the endoplasmic reticulum decreases, cells mediate the influx of extracellular Ca^2+^ ions [121]. During MI, activated B cells secrete various cytokines and chemokines that affect the course of inflammation [122]. B lymphocytes mainly produce antibodies, which can increase myocardial tissue destruction during infarction. Blocking IgM has been shown to significantly reduce ischemic damage [123].

## 4. Application of Ion Channel Therapy in MI

### 4.1. Drugs Targeting Ca^2+^ Channels

**Figure 3:**
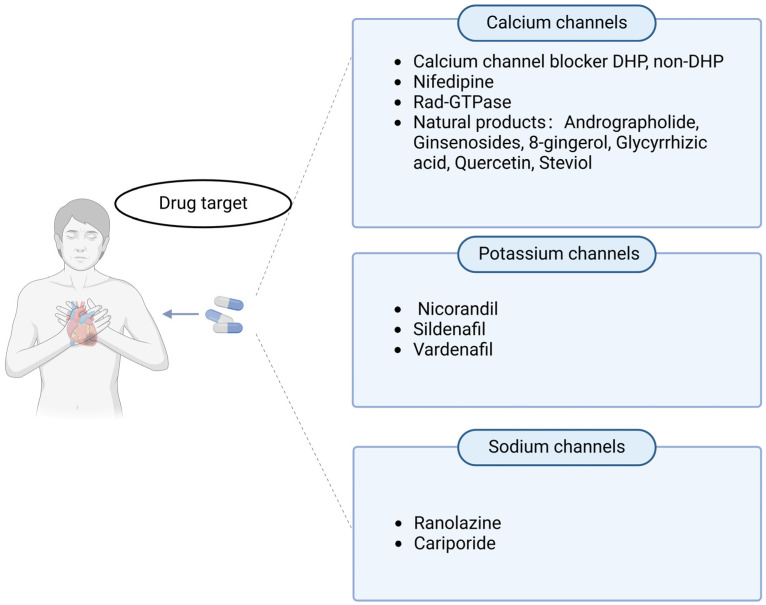
Drugs for ion channel therapy after MI. DHP: dihydropyridine; non-DHP: non-dihydropyridine.

The commonly used Ca^2+^ channel blockers (CCBs) can be divided into two categories: dihydropyridine (DHP) and non-dihydropyridine (NDHP) (Figure 3). Compared with non-DHP, DHP has a shorter half-life and may cause reflex tachycardia [124]. However, non-DHP is more effective in reducing the force and rhythm of myocardial contraction [125]. In the 1990s, short-acting DHB-type CCBs were commonly used to treat myocardial infarction. However, it was later discovered that they could activate sympathetic reflexes, causing tachycardia and hypotension, which could worsen myocardial ischemia [126,127]. As a result, this type of CCB is not allowed for use in patients with myocardial infarction. In later clinical trials, it was found that the combination of long-acting DHP-type CCB amlodipine and angiotensin-converting enzyme inhibitors was more effective in high-risk hypertensive patients, including those with a history of myocardial infarction [128]. NDHP-CCB is less selective than DHP-CCB in peripheral blood vessels and does not cause reflex sympathetic nerve activation. Percutaneous coronary intervention (PPCI) is the preferred treatment for ST-elevation myocardial infarction. Despite the restoration of coronary blood flow after PPCI, impaired myocardial perfusion associated with adverse clinical outcomes is often observed. To address this issue, the NDHP-CCB verapamil has been tested as an adjunctive treatment for PPCI, but its long-term effects still require further clinical research [129]. Non-DHP CCBs must not be used in patients with heart failure or marked bradycardia because of their cardioinhibitory actions [130].

For patients who have had a myocardial infarction caused by a coronary spasm or spastic ischemic attack, CCB prophylaxis is considered better than secondary prophylaxis because it is believed that such patients are likely to develop angina pectoris [130]. The adverse effects of CCBs include palpitations, headache, hot flashes, edema, gingival growth, and constipation. However, new drugs targeting Ca^2+^ channels have been discovered to relieve MI. Rad-GTPase regulates heart rate by maintaining the β-adrenergic receptor signaling cascade (β-AR) through L-type Ca^2+^ channel (LTCC) [131]. Alpha-interacting domain/transactivator of transcription (AID-TAT) peptide was administered to the L-type Ca^2+^ channel subunits to slow down the inactivation rate of L-type Ca^2+^ current and reduce the opening probability of single-channel current, thereby reducing the infarct size [132]. The extracts of natural products such as transheartolactone, ginsenoside, 8-gingerol, glycyrrhizic acid, quercetin, and steviol can inhibit LTCCs and show cardiac protection in animal models of myocardial infarction [61,133,134,135,136,137].

### 4.2. Drugs Targeting K^+^ Channels

K^+^ channel agonists have been shown to have cardioprotective effects in vitro. They restore myocardial contractility, inhibit contractures, and prevent necrosis [138]. These agonists are also commonly used in clinical practice to reduce MI. For instance, nicorandil plays a protective role in myocardial cells by directly opening mitochondrial ATP-sensitive K^+^ (KATP) channels. This increases K^+^ influx to restore mitochondrial function, promote ATP production, reduce ischemic damage during MI, and prevent myocardial cell apoptosis [139]. The interaction between nicorandil and the sulfonylurea receptor (SUR) subunit opens the KATP channels, increasing inward K^+^ flow to restore mitochondrial function and promote ATP generation. K^+^ is a crucial component of mitochondrial ion homeostasis, participating in regulating ROS synthesis and the modulation of the mitochondrial matrix volume. Maintaining mitochondrial volume homeostasis is essential for preserving vesicle integrity during membrane transport with high variations in ions and water, which is crucial for ATP generation [140]. Additionally, it helps in preventing the occurrence of ventricular arrhythmias and improving survival rates during cardiac I/R [141].

Post-ischemic conditioning (I-Post) with three cycles of brief ischemia (30 s) followed by reperfusion (30 s) is more in line with the clinical protocol for the onset and treatment of acute MI. I-Post combined with nicorandil treatment provides effective cardiac protection against ischemia/reperfusion (I/R) injury in diabetic myocardium by activating the PI3K/Akt signaling pathway [142]. Nicorandil upregulates nucleolin expression, subsequently promoting autophagy, and then modulates the TGF-β/Smad signaling pathway, alleviating myocardial remodeling after MI [143]. In addition, pretreatment with nicorandil enhances cardiac repair post acute MI (AMI) by upregulating miRNA-125a-5p in mesenchymal stem cell-derived exosomes (MSC-exo). This upregulation inhibits the TRAF6/IRF5 signaling pathway, promoting M2 macrophage polarization and significantly improving cardiac repair outcomes after AMI [144]. An analysis of 300 STEMI patients who underwent primary percutaneous coronary intervention (PPCI) for the first time, from January 2020 to December 2022, revealed that thrombus aspiration combined with nicorandil leads to superior improvement in cardiac function [145]. A total of 140 patients from the Rawalpindi Institute of Cardiology were enrolled in the study. The use of nicorandil in patients with ST-elevation MI (STEMI) can prevent reperfusion injury, thereby reducing the risk of complications after percutaneous coronary intervention [146]. The study included 5504, 1674, and 3923 patients treated with a combination therapy of nicorandil and trimetazidine. At the 3-year follow-up, the incidence of major adverse cardiovascular events (MACEs) was lower in the combination therapy group. Therefore, the combination of nicorandil and trimetazidine may serve as an effective and potential treatment strategy [147]. The accumulation of toxic metabolites of nicorandil leads to epithelial proliferation and subsequent tissue ulceration [148].

Sildenafil and vardenafil can reduce the MI area and the inhibition of cell necrosis and apoptosis by opening and activating, respectively, the mitochondrial KATP (mitoK ATP) and K–Ca channels [149]. Sildenafil and vardenafil are representative drugs of PDE type 5 inhibitors (PDE5Is), which prolong the physiological effects of NO/cGMP signaling in tissues through the inhibition of cGMP degradation [150]. PDE5Is have been shown to reduce the size of myocardial infarction and inhibit ischemia-induced ventricular arrhythmias [150]. Sildenafil appeared to prolong the ischemia/angina threshold compared to placebo, and PDE5i users have lower mortality post MI [151]. The CYP3A system is the primary metabolic pathway for sildenafil, vardenafil, and tadalafil. CYP2C9, CYP2C19, and CYP2D6 are also involved in the metabolism of sildenafil, and CYP2C9 is involved in the metabolism of vardenafil [152]. The pharmacokinetics of sildenafil are influenced by age as well as renal and hepatic impairment. For patients with severely impaired renal or hepatic function, consideration should be given to a lower starting dose [153].

### 4.3. Drugs Targeting Na^+^ Channels

Ranolazine is a potent late Na^+^ current inhibitor. It intervenes at an important step in the pathophysiology of ischemia by preventing intracellular ion dysregulation. Ranolazine was originally thought to exert its antianginal effects by regulating the metabolism of free fatty acids, but this was only seen when its serum concentrations were higher than those used clinically [154,155]. The drug selectively inhibits late INa without affecting the rapid Na^+^ current responsible for the upstroke of the action potential, particularly in M cells and Purkinje fibers. However, in healthy non-ischemic, non-failing myocytes, where the contribution of late INa is minimal, the drug does not measurably affect the cardiovascular performance at therapeutic plasma concentrations. In patients with chronic angina and demand-induced ischemia, ranolazine has the potential to partially mitigate the consequences of cellular hypoxia during transient myocardial ischemia by reducing excessive late Na^+^ influx. This reduction leads to a decrease in Ca^2+^ overload and ultimately reduces the associated increase in left ventricular wall tension [156].

The Na^+^–hydrogen exchange (NHE) inhibitor cariporide has been shown to have therapeutic effects on acute MI. In patients with acute MI, 40 mg of intravenous cariporide after percutaneous coronary intervention can reduce the myocardial end-systolic volume, increase the ejection fraction, and improve regional wall motion abnormalities within 3 weeks [157]. In patients with acute ST-segment elevation MI, the administration of an NHE-1 inhibitor before reperfusion therapy can reduce the incidence of arrhythmia [158].

### 4.4. Basic Ion Channel Physiology and Clinical Applications

Ischemia/reperfusion injury is a complex cascade of molecular reactions that can lead to harmful cellular damage and organ dysfunction. Targeting a single reaction mechanism with specific therapy may not provide sufficient value in multifactorial clinical cases [159]. If the goal of cardioprotection research is truly translation, it must evolve from reductionist models to real-world scenarios [160]. Many cardiovascular risk factors, comorbidities, and concurrent medication therapies can influence the severity of ischemia/reperfusion injury (I/R), such as aging, diabetes, and hyperlipidemia [161]. However, most studies on cardioprotection have been conducted in I/R animal models without comorbidities [162]. When designing preclinical studies to identify and validate targets for cardioprotective drugs, as well as clinical trials, it is imperative to consider the presence of cardiovascular risk factors and concomitant medications. This approach is expected to maximize the success rate of developing rational cardioprotective therapy strategies for the majority of patients with multiple risk factors.

K201 is a 1,4-benzothiazepine derivative that inhibits the Na^+^ current (INa) in a voltage- and frequency-dependent manner. Additionally, K201 mildly blocks Ca^2+^ current (ICa) and inward-rectifier K^+^ current (IK1) [163]. K201 exhibits protective effects against myocardial ischemia and catecholamine-induced myocardial injury [164]. Ca^2+^ channel blockers (CCBs) bind to voltage-dependent Ca^2+^ channels in the cell membrane, inhibiting the influx of Ca^2+^ ions into the cell. Increasingly, both basic and clinical trials have confirmed the efficacy of these drugs when used alone or in combination with other medications, for example, the combination therapy of benazepril (Cibacen) and amlodipine. However, further research is still needed in this field [130].

Ion channels represent a large membrane protein superfamily critical for physiological functions in both excitable and non-excitable cells. While patch-clamp electrophysiology remains the gold standard for screening ion channel inhibitors, this approach is associated with technical challenges, high costs, and slow throughput [165]. With the advancement of cryo-electron microscopy (cryo-EM) technology, the structures of ion channels are becoming increasingly elucidated. Utilizing computer-aided drug design methods allows for the identification of novel lead compounds, thereby accelerating the pace of ion channel drug discovery [166]. Using mass spectrometry for high-throughput proteomic analysis provides valuable insights into the functional interactions of ion channels within macromolecular signaling complexes in vivo [167]. Moreover, the rapid advancement of machine learning algorithms, such as deep learning (DL), which enables the construction of complex and flexible models based on data, greatly facilitates the clinical translation of ion channel drugs [168].

Targeting ion channels is not always beneficial. Due to the unintended targeting of ion channels by drugs, the development of acquired channelopathies or hazardous side effects may occur (such as an increased risk of arrhythmias or delayed ventricular repolarization) [169].

## 5. Perspectives and Conclusions

Ion channels are widely distributed in the human body and represent attractive targets for drug development. In the treatment of cardiovascular diseases, ion channel inhibitor drugs are used to block specific channels and transport proteins to redistribute ions and restore the normal function of the heart [170]. However, at present, few well-developed and effective ion channel drugs have been found for the clinical pharmacological treatment of MI [171].

In existing research, the small-molecule drugs targeting ion channels exhibit poor targeting and significant off-target toxicity. In addition, due to the presence of multiple transmembrane proteins, the extracellular region of ion channels is very small, and it is difficult to develop antibodies against ion channels, which poses a great challenge for the development of targeted drugs. With the advances in computer modeling and structural biology technology, ion channels in the heart are expected to be accurately characterized and analyzed. Resolving the complex molecular mechanism behind the regulation of ion channels poses a further challenge.

To this end, X-ray crystallography and cryo-electron microscopy for improved structure-based drug design and the use of genomic and proteomic associations with MI to identify new therapeutic targets are creating significant opportunities for the development of ion channel drugs. X-ray crystallography and cryogenic electron microscopy can help to provide information about the structural biology of ion channels and related proteins, which has always played an important role in all stages of preclinical drug development, from the optimization of lead compounds to the identification and design of drug targets [172,173]. Genomics is the study of all genes in a certain biological unit, including chromosomal DNA, all forms of RNA, and transcriptional variants. The completion of the sequencing of the genomes of humans and other organisms will help identify many potential new drug targets in diseases and is expected to lead to the development of novel ion channel drugs against them [174]. Furthermore, proteomics is the direct analysis of protein content, modifications, and interactions. Genomics and proteomics can complement each other to have an impact on the discovery and application of ion drugs to identify and validate therapeutic targets and biomarkers, thus illustrating drug action mechanisms and determining clinical effects [175].

Furthermore, with the advances in computer modeling, the ion channels in the heart are expected to be accurately characterized and analyzed. Computational models have become important tools for understanding the biophysical mechanisms of the ventricular action potential, relating changes in gene/protein expression to alterations in action potential and ion transient; this is important in the study of the pharmacological effects of ion drugs [176].

With the increasing research on ion channels and the development of biomedical technology, the discovery of ion drugs and the expansion of therapeutic strategies are expected to lead to breakthroughs in the field of therapeutics for MI.
